# Development and validation of the care challenge scale in family caregivers of people with Alzheimer's disease

**DOI:** 10.3389/fpubh.2022.921858

**Published:** 2022-07-28

**Authors:** Hamid Sharif Nia, Erika Sivarajan Froelicher, Lida Hosseini, Mansoureh Ashghali Farahani, Sima Hejazi

**Affiliations:** ^1^School of Nursing and Midwifery, Mazandaran University of Medical Sciences, Sari, Iran; ^2^Department of Physiological Nursing, School of Nursing, University of California, San Francisco, San Francisco, CA, United States; ^3^Department of Epidemiology & Biostatistics, School of Medicine, University of California, San Francisco, San Francisco, CA, United States; ^4^School of Nursing and Midwifery, Iran University of Medical Sciences, Tehran, Iran; ^5^Nursing Department, Bojnurd Faculty of Nursing, North Khorasan University of Medical Sciences, Bojnourd, Iran

**Keywords:** family caregiver, Alzheimer, validity, reliability, challenge, scale, questionnaire

## Abstract

**Background:**

Alzheimer's disease (AD) is a progressive and debilitating disorder that strongly affects people with AD and their families. The changes in signs of the disease and its treatment lead to many challenges in people with AD that affect the performance and the ability of caregivers, their social life, and physical, emotional, and psychological aspects of caregivers' health. Therefore, this study was designed to develop and validate the Care Challenge Scale (CCS) for family caregivers of people with AD in the care context of Iran.

**Method:**

This is a cross-sectional study, and the primary scale was based on 14 semi-structured interviews with family caregivers of Iranian people with AD. In the next phase, the psychometric features were assessed, including the face validity (qualitative and quantitative), content validity (qualitative and quantitative), item analysis, structural validity (exploratory and confirmatory factors), and construct validity (convergent and discriminant validity). Finally, the reliability was assessed using internal consistency (Cronbach's alpha, McDonald's omega coefficient, and the average inter-item correlation), stability (intraclass correlation coefficient), and absolute reliability.

**Results:**

Totally, 435 Iranian family caregivers filled out online questionnaires, with a mean age of 50.26(±13.24) years. Based on the results of the qualitative phase, an item pool was generated with 389 items, and after deleting overlapping and unrelated items, the CCS with 14 items was created. The results of the quantitative phase showed that the CCS consists of two factors with 10 items each, which are named effective role-play challenge and lack of social–financial support, and they explained 42.23% of the total variance. Furthermore, the results of confirmatory factor analysis showed a good fitness of the scale structure model, and it had convergent and discriminant validity. The reliability indexes showed this scale has internal consistency and stability.

**Conclusion:**

The most care challenge among Iranian family caregivers of people with AD is effective role-play challenges and lack of social–financial support. The scale as designed has good validity, internal consistency, and stability that can be used by therapists, nurses, and researchers for the assessment of the challenges of this population.

## Introduction

Alzheimer's disease (AD) is a chronic, progressive, and debilitating brain disorder that is associated with profound effects on memory, intelligence, impairment in speech, motor activity, cognition, and general dysfunction (1). Alzheimer's Disease International (ADI) has reported that approximately 35.6 million people with dementia live worldwide in 2010, and this will double every 20 years; the prevalence will increase from 57.4 million cases globally in 2019 to 152.8 million cases in 2050 (2). Studies show the prevalence of dementia is influenced by cultural and socioeconomic factors and varies widely across countries. The increase in dementia is greater in developing countries, with 58% of people with dementia in developing countries, and this rate is projected to reach 71% by 2050 (3, 4). A study on AD in Iranian elders shows that there are more than 700,000 people with AD in Iran, that is, one of every 11.5 persons (5). It is estimated that 8 to 10% of the Iranian elders will be affected by this disease in the next two to three decades (6, 7).

These people become dependent on others to meet their needs due to cognitive and behavioral disorders, and this dependence on self-care increases over time (8, 9). Studies have shown that informal caregivers provide more than 81% of the care needed by patients with AD (10). In Iran, it is estimated that seven of 10 patients with AD are cared for at home (11, 12).

Inter-cultural studies show that the type of caregivers for these patients differs between Western and eastern countries (13). Iran, with a large number of people with AD, has two basic values, namely, altruism and strong family ties that result in greater commitment to relatives, especially if they are family members who experience an illness (14). In Asian countries, where community support services and resources needed by these people are lacking, families have become the first line of support (15, 16). Family members are forced to take full responsibility of caring for a person with dementia. While Western caregivers receive formal support. The Iran Alzheimer Association (IAA) is the only voice for people with various forms of dementia and is mainly engaged in the following activities: raising public awareness, clinical and rehabilitative activities, counseling, and education for patients and caregivers. As a result, the families and relatives are the main source of caring for these patients in Iran (14).

Since AD is a progressive and debilitating disorder, the people with AD and their families are constantly affected by the changes resulting from the disease and its treatment. These changes lead to challenges and needs that people with AD are affected with; thus, caregivers need to learn how to perform activities of daily living in people with AD (12, 17). The most important challenges that these caregivers experience during caregiving include physical, psychological, emotional, social, and financial challenges that, if not addressed properly, may lead to complications for the caregivers and the patients (16). Therefore, AD affects not only the patients but also the caregivers. According to the ADI, approximately half of the caregivers experience health, work, and social problems each year, which are referred to as “the caregiver burden” (18). Caregiver burden is defined as “a multidimensional response to the physical, psychological, emotional, social, and financial stressors associated with the care experience provided” (19). Caregiver burden is classified into the objective burden and subjective burden. The objective caregiver burden arises from spending time caring for and providing physical care to the patients such as helping the patients meet their personal needs and assisting with financial problems arising from care. While the cause of subjective caregiver burden in the caregivers is related to their perception of their ability to master dementia care, resource management, and gaining satisfaction from their caregiver role (9). This caregiver burden makes caregivers highly susceptible to a variety of problems. These problems include social isolation, deterioration in physical health, cardiovascular disease, mental health problems such as depression, lower levels of subjective wellbeing, anxiety, overuse of medication, and increased need for medical services (20). Many factors have been reported to affect the severity and amount of caregiver burden perceived by family caregivers of people with AD. These can be classified into two categories: patient-related factors such as disease severity, behavioral and psychological symptoms of dementia (BPSD), and disease duration, and caregiver community factors including kinship, gender, coping strategies, individual values and beliefs, community, culture, and the number of support resources available in the community (21–23). Individual values and culture greatly impact the motivation of caregivers to use resources or support and coping styles to care for people with AD, and as a result, the perceived intensity and amount of caregiver burden felt and its complications may vary (24).

Research has shown that the challenges of family caregivers is the main factor that adversely affects functional, social, emotional, psychological, and financial aspects of caregivers (25). Therefore, prevention and reduction of the challenges of caregiving can significantly affect the caregivers. For this reason, a suitable scale is needed to quantify the important challenges that caregivers encounter.

Numerous tools have been developed to measure caregiver burden (26). One of the well-known tools primarily developed for assessing the burden on caregivers of people with dementia is the Zarit Burden Interview. It consists of 29 items and no subscale (27). Also, Taemeeyapradit et al. (28) developed a scale for assessing burden on caregivers of people with dementia with 18 items and three subscales (28). In Iran, Abdollahpour et al. (29) developed a Persian language caregiver burden scale based on a literature review and expert opinion for caregivers of people with dementia (29).

The causes of caregiver burden are physical, psychological, social, and financial and account for the majority of the challenges that these caregivers face. To our knowledge, most studies have focused exclusively on describing caregiver burden and side effects from the caregiver perspective but have not addressed the important challenges of the caregivers. However, the lack of an appropriate scale to measure main caregiver challenges during caring has resulted in the lack of quantifiable data. Since respect for elders is a very important value in Iranian culture and these caregivers do not have enough support services, they face many challenges while caring for people with AD. Therefore, the present study aims to develop and evaluate the psychometric properties of the Care Challenge Scale (CCS) in family caregivers of people with AD in the care context of Iran.

## Methods

### Design

A cross-sectional design was used to develop and validate the CCS in family caregivers of people with AD. A two-phase process was used: (1) the first phase (qualitative phase) consisted of semi-structured interviews with the target population for item generation, and (2) the second phase (quantitative phase) consisted of assessment of psychometric features of the developed scale.

### First phase: Qualitative phase (item generation)

In order to clarify the concept of the main care challenges in the Iranian context based on the experiences of the target community, 14 semi-structured interviews were held with Iranian family caregivers. Their mean age was 54.57 years. The study was conducted between November 2020 and February 2021. Of the 14 participants, nine participants were daughters of patients, two participants were sons of a patient, and three participants were the spouse of patients. Their educational levels were as follows: eight had an academic education, four had diplomas, and two had elementary education. Overall, six participants were employed, and eight were not employed for various reasons such as retirement or leaving their job to care for a family member.

A purposeful and snowball sampling method was used to select these participants. The inclusion criteria in this phase were as follows: the family caregivers who were a member of the patients' family, friends, or relatives who were responsible for caring for the patients; family caregivers whose patients had moderate to severe AD and depended on caregiving for activities of daily living; and those who had the ability to express and recall their experiences. The interviews lasted between 30 and 90 min. Totally, 12 interviews were conducted at the clinic in one hospital in Tehran, and two interviews were conducted at the caregivers' homes. The sample size in this phase was based on data saturation (absence of new data). In this study, after 13 interviews, the data were saturated, and the last interview was conducted to ensure saturation.

The interview guide contained open-ended questions based on the study objectives, which were formulated after consulting the research team. Moreover, based on the data, exploratory questions were asked to the participants to deepen our understanding of their experiences. The interview guide is shown in Table 1. Examples of exploratory questions that were used to guide the interviews included the following: “Can you explain more about this?”, “Can you give an example?”, “When you say.... What do you mean?”

**Table 1:** Interview guide.

Introductory questions	How long has your elderly person had Alzheimer's disease? How long have you been caring for the patient? Can you talk about the care you gave to your People with Alzheimer's?
Challenge of caring	Have you ever been tired of caring? What bothers you about your care? What are the most important challenges you faced while caring?
Demographics	What is your marital status? Do you live with the patient? How old are you?
Final question	Do you have anything else to say about your challenges while caring from your patient?


After each interview, the recorded interview was transcribed. The written text was carefully studied several times by the first researcher and coded using guided content analysis (30). In order to facilitate the coding process, we used MAXQDA software ver.10. At the end of this stage, 389 initial codes were extracted, and they were categorized into three themes. Based on the result of this phase and the extracted codes, an item pool of 389 items was created during frequent meetings of the research team, all of which were carefully studied. Duplicates, overlaps, and similarities of the items were checked, and some items were merged or deleted. Therefore, the total number of items was reduced to 50 and then to 14 items. Finally, the basic form of the CCS had 14 items with five-point Likert response options (1 = never, 2 = rarely, 3 = sometimes, 4 = often, 5 = always) for the care context in Iran were designed based on the remaining codes. An electronic form of the questionnaire was created using Google Forms, and the data were collected online in different steps of psychometrics.

### Second phase: Psychometric evaluation (item reduction)

During this stage, the initial scale was designed based on the qualitative phase and was evaluated in terms of the psychometric properties using face, content, and construct validity, as well as reliability. At each stage of the psychometric evaluation, inappropriate items were removed according to the criteria of that stage. The sample size at each stage was different (which is explained separately in each stage).

### Face validity

Face validity was checked *via* qualitative and quantitative approaches. To perform qualitative face validity, 10 family caregivers were asked to evaluate items in terms of the level of difficulty or ambiguity in answering the questions, and based on their opinion, the items were edited by the research team. During quantitative face validity assessment, the impact score of each item was calculated by asking the same 10 family caregivers to assess the suitability of each item using a five-point Likert response (5 = it is completely suitable, 4= it is suitable, 3 = it is almost suitable, 2 = it is a little suitable, 1 = it is not suitable at all). The impact score formula included the following: the impact score = frequency (%) × suitability; an impact score of > 1.5 is considered acceptable (31).

### Content validity

Like face validity, content validity was also evaluated using qualitative and quantitative approaches. In order to do qualitative content validity, we asked 12 experts (in nursing, psychology, instrument development, and gerontology) to assess each item. The content experts endorsed the items in terms of grammar, wording, item allocation, and scaling. Based on their opinion, some items were edited by the research team. During the evaluation of the quantitative content validity of the scale, the content validity ratio (CVR), content validity index (CVI), and modified kappa coefficient (K) were calculated. In the CVR, we asked the same 12 experts to evaluate how essential each item was using a three-point Likert response (1 = not essential, 2 = useful but not essential, 3 = essential), and an acceptable CVR was based on the Lawshe formula (21) (for 12 experts, it is 0.56) (32). At this stage, all of the items were acceptable (CVR > 0.56). In the CVI, we asked 11 different experts to evaluate the relevance of each item using a dichotomous response (1 = relevant, 0 = irrelevant). A chance effect was eliminated by calculating the modified kappa (K), where K > 0.74 was considered excellent, and a score of 0.60–0.74 was considered good (31).

### Item analysis

Possible problems with the items before entering the construct validity stage were investigated using item analysis and calculating the corrected item total correlation. During this stage, at first, we designed the online form of the questionnaire and then we sent its link to 32 family caregivers (the mean age of participants was 52.02 ± 13.91). Items whose correlation coefficient was <0.32 between cases were deleted (31).

### Construct validity

#### Participants

The sample was Iranian family caregivers such as family members, relatives, and friends of people with AD who provided care and was willing to participate in the study. Because the data were obtained using an electronic form of the questionnaire through social networks such as Telegram and WhatsApp, samples were selected if they were able to use these social networks. The sample size for a factor analysis study was based on the rule of thumb, that is, 10 subjects per item are considered suitable (31). Thus, the sample of 435 family caregivers was sufficient for the two stages [(210 for evaluating exploratory factor analysis (EFA) and 225 for evaluating confirmatory factor analysis (CFA)].

#### Measures

**Figure 1:**
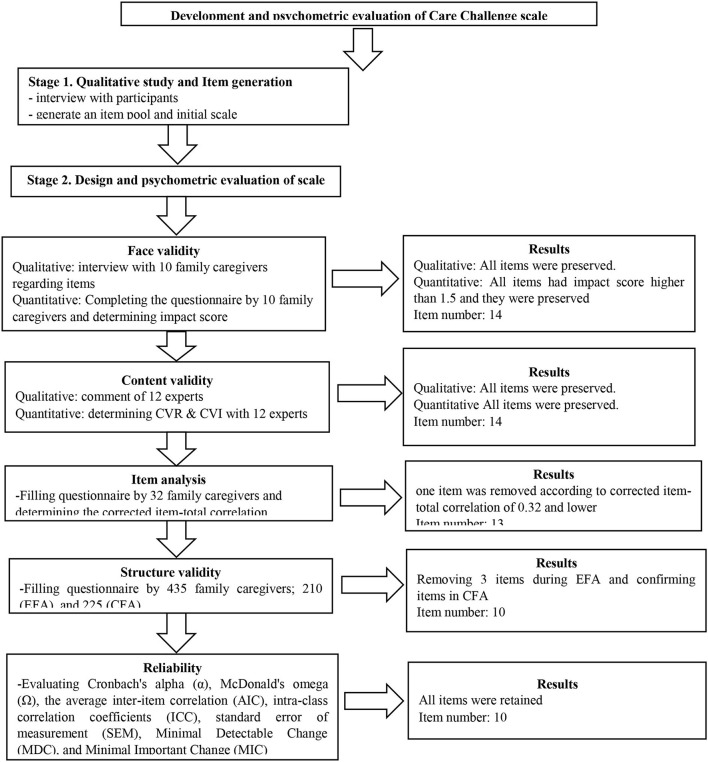
Production phases of the care challenge scale.

Data were collected at this stage in two parts. The first part included demographic characteristics such as age, sex, marital status, education level, employment, lifestyle (independent, living with a patient), and relationship to the patient. The second part included the CCS with 13 items and five-point Likert response options (1 = never, 2 = rarely, 3 = sometimes, 4 = often, 5 = always). The details of the production phases of CCS (reduction and creation) are shown in Figure 1. Data were gathered online and extracted into an Excel file. Therefore, the online questionnaire was created *via* Google Forms, and its URL link was sent to participants by email or through social networking applications such as Telegram channel or WhatsApp.

EFA, CFA, and convergent and divergent validity were used. At first, EFA was performed with the maximum-likelihood exploratory factor analysis (MLEFA) approach. EFA is a method for analyzing variance between several dependent variables based on their description in terms of a small number of latent variables (factors). EAF seeks to simplify complex data by describing them in terms of a smaller number of variables. EFA also allows for latent constructs to be better understood and explain more logically the items that reflect them (33). EFA assumed that there is a relationship and correlation between latent variables and a change in one latent variable affects another variable (33). The caring challenges is a concept in psychology and social sciences, and it seems that there is a relationship between its latent variables. Therefore, exploratory factor analysis was used in this study.

In psychological studies, as long as there is no strong evidence that there is no relationship between the latent factors, it is recommended to use the oblique rotation method to extract the factors. In social sciences, it is expected that there are relationships between factors. Therefore, if the factors are related, the orthogonal rotation will cause a loss of valuable information (31). Therefore, the oblique rotation method was used in this analysis.

In order to evaluate the quality of responses and the quality of the samples, the Kaiser–Meyer–Olkin (KMO) and Bartlett's tests were calculated. The KMO values higher than 0.9 were interpreted as excellent. The number of suitable extraction factors was determined using Horn's parallel analysis and the exploratory graph analysis approach (31), where a value of 0.3 was set for the correlation between the factors. Promex rotation is the most common rotation used in humanities, and it is used to insert specific items for each factor. Horn's parallel analysis provides more accurate results for determining the number of main scale factors. It creates a random score matrix that has exactly the same rank and type of variables as is in the data set. Comparison of the actual values of the randomly generated matrix determines the correct number of factors and has more variance than the components of the random data (34).

The number of items for each latent factor was determined by accounting for the factor loading. The factor loading formula included the following: CV = 5.152 ÷ √ (*n* = 2), where CV is the number of extractable factors, “N” is the sample size, and a factor loading of 0.36 is acceptable for retaining the item in the factor (33).

The factor structure obtained by EFA was examined by CFA. The maximum-likelihood method was also used. The most common goodness-of-fit indicators of the proposed model were based on their accepted threshold using the chi-square (χ^2^) test, chi-square/degree-of-freedom ratio (χ^2^/df) <4, comparative fit index (CFI) >0.90, incremental fit index (IFI) >0.90, normed fit index (NFI) >0.90, Tucker–Lewis index (TLI) >0.90, relative fit index (RFI) >0.90, root mean square error of approximation (RMSEA) <0.08, Parsimonious Normed Fit Index (PNFI) >0.50, and Parsimonious Comparative Fit Index (PCFI) >0.50 (33).

#### Convergent and discriminant validity

Convergent and divergent validity of the structure were measured by Fornell and Larker's (35) approach (35) based on the following parameters: the average variance extracted (AVE), maximum shared squared variance (MSV), and composite reliability (CR). To confirm convergent validity, AVE must be > 0.5 and CR > AVE. To confirm divergent validity, MSV must be < AVE (36). Furthermore, discriminant validity was evaluated using a new approach, the heterotrait-to-monotrait ratio (HTMT) criteria. A value of <0.85 was considered evidence of discriminant validity (36).

#### Reliability

Cronbach's alpha, McDonald's omega coefficient (Ω), and the average inter-item correlation (AIC) were used to determine the internal consistency of the scale. Therefore, the coefficient's α and Ω values >0.7 and AIC between 0.2 and 0.4 were considered acceptable (37). Also, CR and maximum reliability (Max H) >0.7 of the structural education model were used as criteria to determine reliability (37). The intra-class correlation coefficients (ICCs) were used to determine the stability with a two-week interval in 30 family caregivers (38). Furthermore, the absolute reliability was evaluated using the standard error of measurement (SEM) using the following formula: (SEM = SDPooled × √1 – ICC). Finally, the responsiveness and interpretability of CCS were evaluated by counting the minimal detectable change (MDC) using the following formula: MDC95 = SEM × √2 × 1.96; the minimal important change (MIC) was calculated using the following formula: MIC = 0.5 × SD of the Δscore, respectively, and ceiling and floor effect.

#### Multivariate normality and outliers

Univariate and multivariate outliers were evaluated using distribution charts and Mahalanobis distance *p* < 0.001. Furthermore, univariate normality and multivariate normality distributions were checked by skewness (±3), kurtosis (±7), and Mardia's coefficient (>8), respectively (39).

### Data analysis

Data were analyzed using SPSS/AMOS_26_, SPSS R-Menu_2.0_ and JASP_0.16.2.0_.

### Ethical consideration

The Ethics Committee of the Mazandaran University of Medical Sciences assessed the protocol of this study and approved the study (IR.MAZUMS.REC.1401.13880). Ethical points observed in the item generation phase were as follows: (1) assuring participants that their information is confidential and (2) obtaining written and oral permission from participants for audio recording. In the validation stage of the scale, the necessary information of the study including the purpose of the study, the code of ethics of the study, the number of questions, and the characteristics of the research was mentioned in the first part of the online questionnaire form.

## Results

### Demographic characteristics of participants

During construct validity, 435 family caregivers, with a mean age of 50.26 (±13.24) years, participated. Most of them were female (50.6%) and married (68.7%). The details of demographic characteristics are given in Table 2.

**Table 2:** Demographic characteristics of participants (*n* = 435).

**Variables**	**Mean±SD**
Age	50.26 ± 13.24
Average h of care per day (h)	7.51 ± 5.51
Duration of the disease (year)	4.65 ±2.52
Sex *n* (%)
Female	220 (50.6)
Male	215 (49.4)
Marital status
Single	92 (21.1)
Married	299 (68.7)
Divorced	14 (3.2)
Widow	30 (6.9)
Education level
Illiterate	11 (2.5)
Less than diploma	30 (6.9)
Diploma	200 (46)
Academic	194 (44.6)
Employment
Unemployed	42 (9.7)
Employed	161 (37)
Housewife	146 (33.6)
Retired	24 (5.5)
Not employment	62 (14.3)
Lifestyle
Independent	262 (60.2)
With patients	173 (39.8)
Relationship with the patient
Daughter	230 (52.9)
Son	57 (13.1)
Wife	37 (8.5)
Husband	20 (4.6)
Friend	34 (7.8)
Relative	57 (13.1)

### Item generation

An item pool was generated with 389 items after deleting overlapping and unrelated items; the CCS with 14 items and a five-point Likert response (1 = never, 2 = rarely, 3 = sometimes, 4 = often, 5 = always) was created and entered to the next phase.

### Item reduction

Based on the result of the impact score, CVR, and modified kappa (K), no items were removed. During the item analysis step, one item was removed, and the CCS with 13 items was entered into the factor analysis step.

### Construct validity

**Figure 2:**
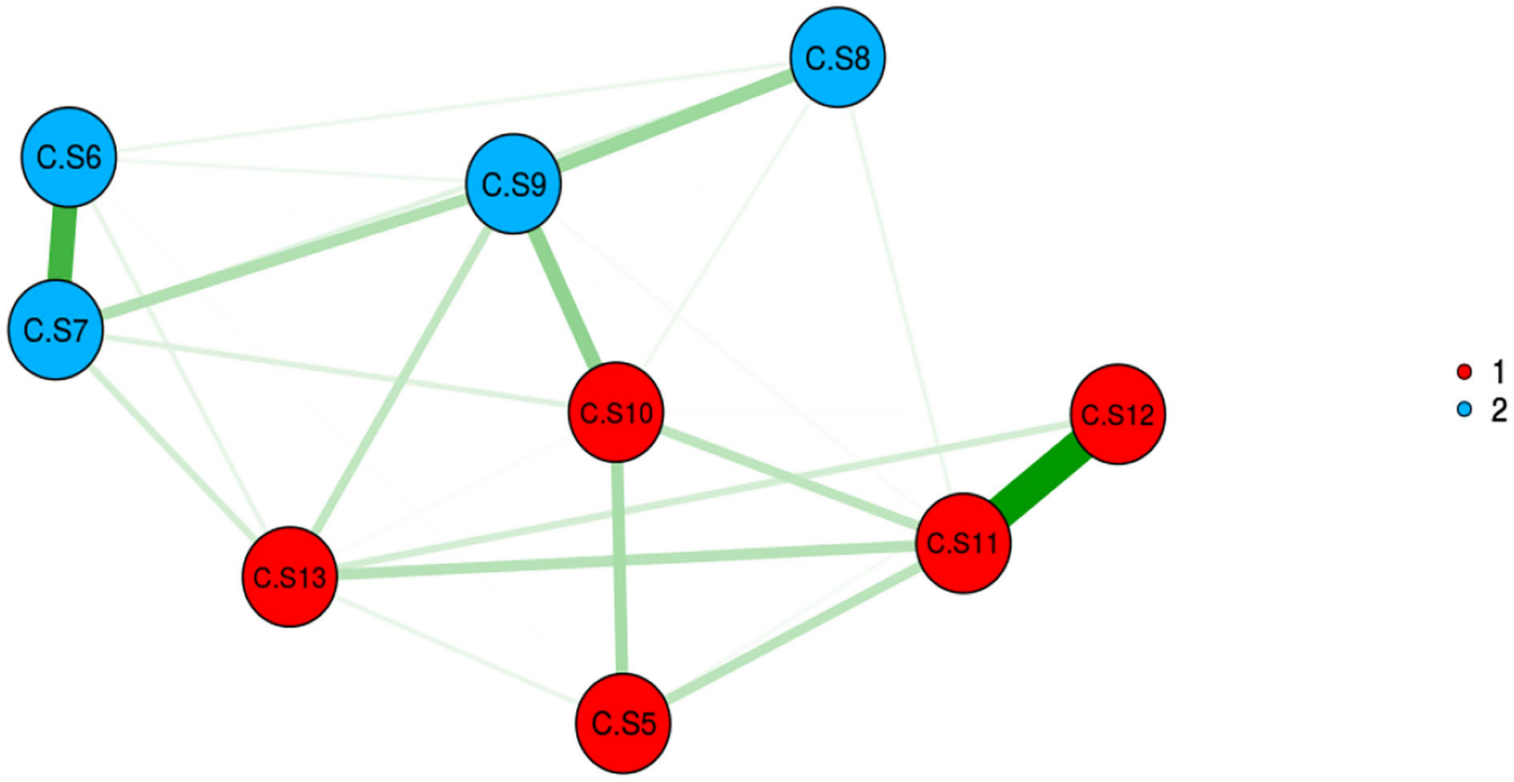
Exploratory graph analysis. *CS, challenge scale.

**Figure 3:**
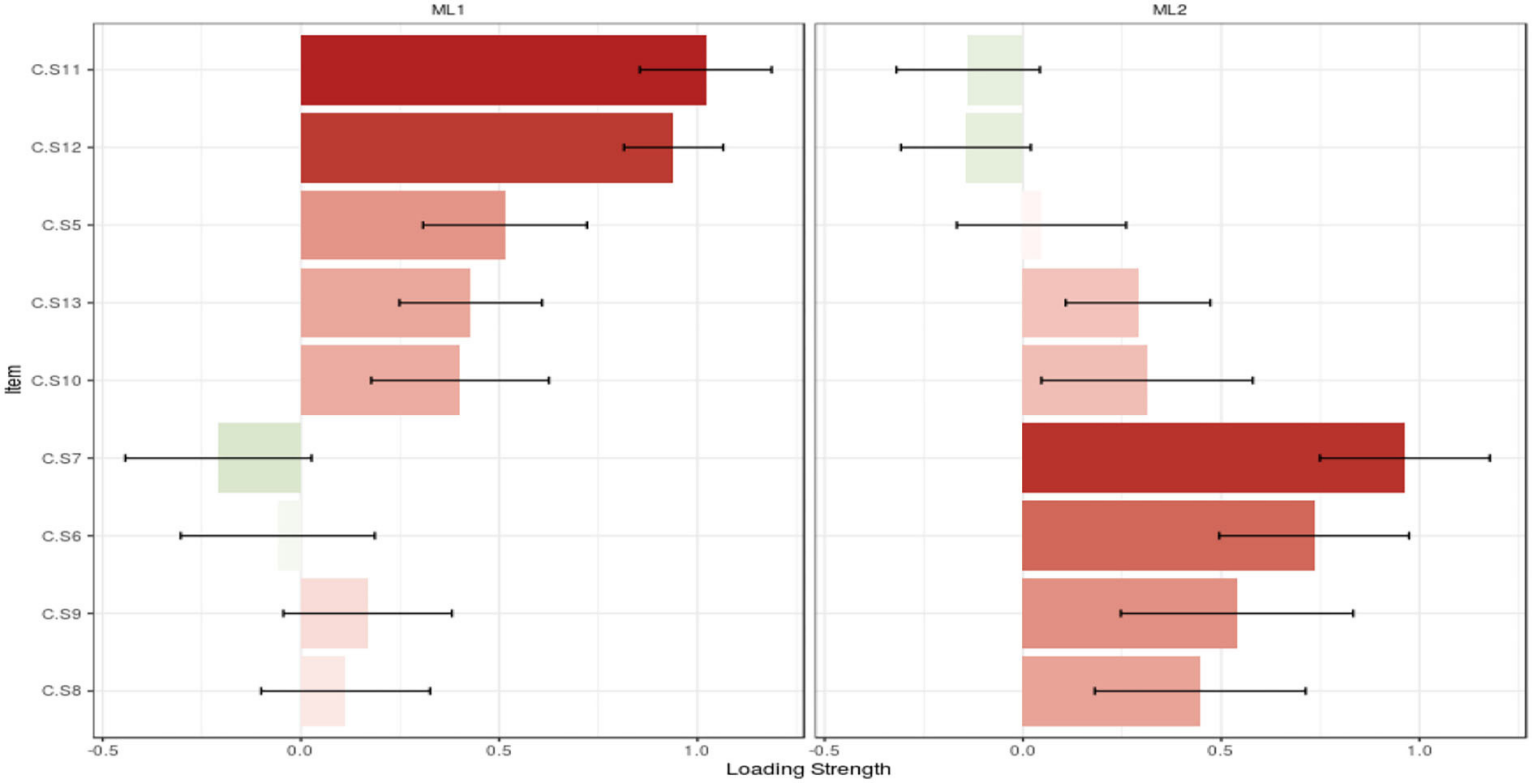
Loading strength of items in factors. *ML1, maximum-likelihood factor 1; ML2, maximum-likelihood factor 2.

**Figure 4:**
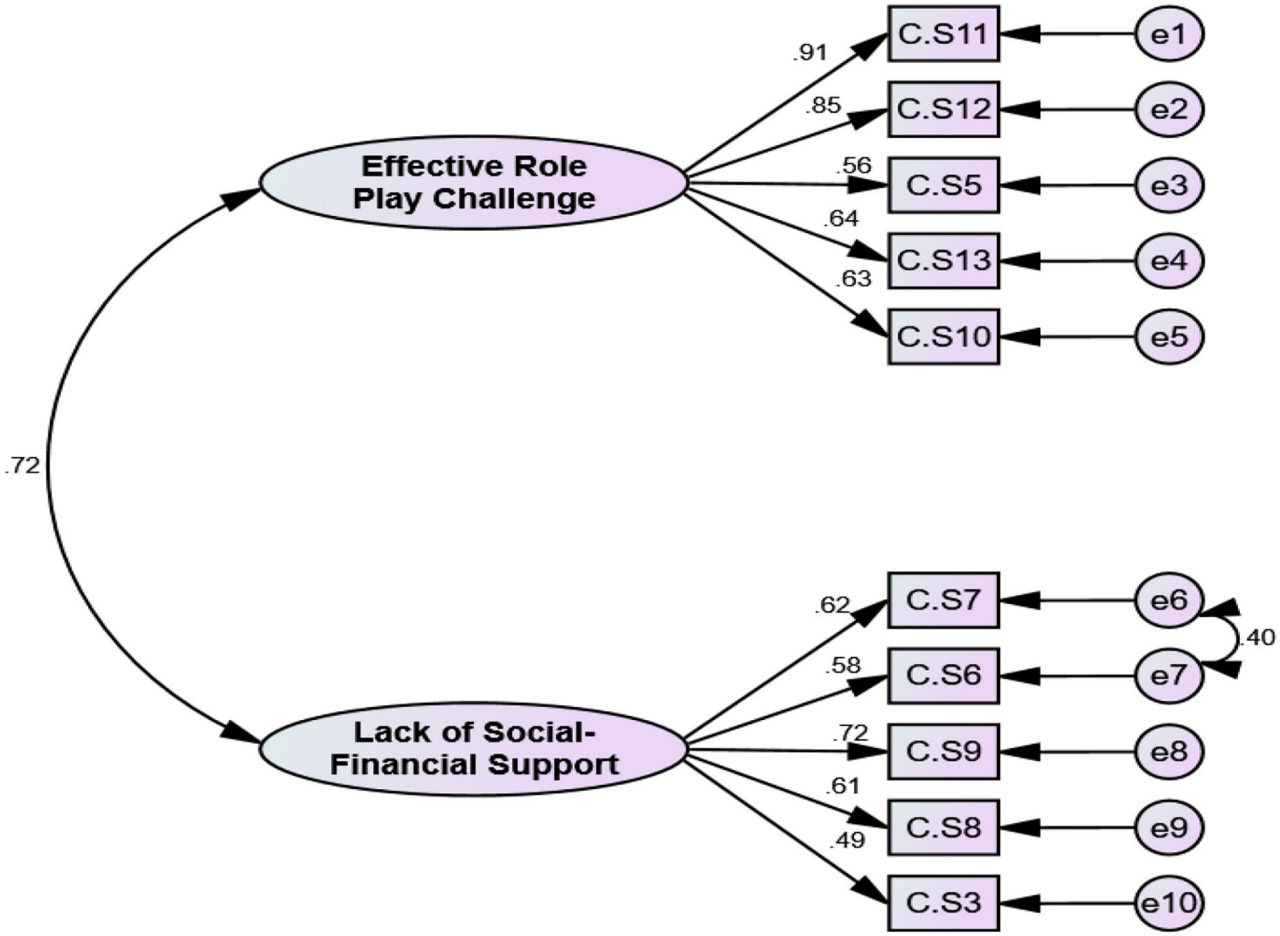
First-order CFA of CCS (*n* = 225). *CS, challenge scale.

The adequacy and suitability of the sample were confirmed based on the results of KMO (0.841) and Bartlett's value of 756.401 (*p* < 0.001). In EFA with Promax rotation, 10 items remained, and they were classified into two factors, namely, F1: with five items, and F2: with five items. These two factors explained 42.23% of the total variance of care challenges in family caregivers of Alzheimer's patients. The details of the results of this step are given in Table 3, Figures 2, 3. The model extracted in EFA was evaluated during CFA, and the results showed this model had good fit indices. Details are provided in Table 4, Figure 4.

**Table 3:** Result of EFA on the two factors of CCS (*n* = 210).

**Factors**	**Q_n_. item**	**Factor loading**	**h^2^**	**λ**	**%Variance**
Effective role play challenge	11. It is difficult for me to control my patient's unusual behaviors.	0.976	0.879	2.350	23.50
	12. It is difficult for me to control my patient's anxiety and worry.	0.891	0.727		
	5. I do not have the ability to communicate properly with the patient.	0.489	0.309		
	13. Due to my patient's unusual behaviors, I have to control his/her social interactions.	0.437	0.448		
	10. Not having enough information about how to care for a patient puts more pressure on me.	0.417	0.428		
Lack of social - financial support	7. The lack of insurance for some aspects of treatment puts me under pressure.	0.857	0.633	1.873	18.73
	6. Taking care of the patient is costly for me.	0.712	0.496		
	9. The lack of proper support centers in the community increases the pressure on me.	0.514	0.433		
	8. Not cooperating those around me increases the pressure of caring for me.	0.450	0.287		
	3. Patient care has affected my job.	0.407	0.207		

* h^2^, communalities; λ, eigenvalue.

**Table 4:** Fit indices of the CFA model after structure modification of the CCS (*n* = 225).

CFI	IFI	TLI	PCFI	RFI	NFI	PNFI	RMSEA	CMIN/DF	*P-value*	Df	χ^2^	Indices
0.929	0.930	0.903	0.681	0.851	0.891	0.653	0.042	2.565	<0.006	33	44.661	CFA model

*DF, degree of freedom; PCFI, Parsimonious Comparative Fit Index; PNFI, Parsimonious Normed Fit Index; CMIN/DF, minimum discrepancy function divided by degrees of freedom; RMSEA, root mean square error of approximation; TLI, Tucker–Lewis Index; and CFI, comparative fit index, IFI, incremental fit index*.

*Fitness indexes, PNFI, PCFI (>0.5). TLI, IFI, CFI, NFI, RFI (>0.9), RMSEA (<0.08), CMIN/DF (<3 good, <5 acceptable)*.

Based on the results of AVE and CR, the first factor had the convergent and discriminant validity, but the second factor did not have these features. It is noteworthy that based on the result of HTMT, two factors had discriminant validity (Tables 5, 6).

**Table 5:** Indices of the convergent, discriminant validity, and internal consistency of CCS (*n* = 225).

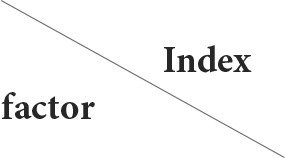	CR	AVE	MSV	MaxR (H)	Alpha	Omega	AIC
factor							
Effective role play challenge	0.848	0.537	0.513	0.905	0.838	0.837	0.515
Lack of social - financial support	0.745	0.372	0.513	0.758	0.765	0.773	0.393

*DF, degree of freedom; CR, composite reliability; AVE, average variance extracted; MSV, maximum shared squared variance; AIC, average inter-item correlation*.

**Table 6:** Results of HTMT of CCS (*n* = 225).

**Dimensions**	**Effective role play challenge**	**Lack of social - financial support**
Effective role play challenge		
Lack of social - financial support	0.765	

These two factors had acceptable internal consistency based on results of Cronbach's alpha, McDonald's omega coefficient, and AIC. The details of these results are shown in Table 4. Also, the ICC score was 0.90, indicating that the scale has strong stability. The absolute reliability was ±2.23, and based on results of MDC, MIC, LOA, ceiling, and floor effects (items were free of these effects), this scale had responsiveness and interpretability features (Table 7).

**Table 7:** Results of stability, SEM, responsiveness, and interpretability (*n* = 30).

	ICC	SD	Mean	SEM	MDC 95%	MIC	LOA
Scale	0.902	7.13	32.46	2.23	6.18	3.56	46.43–18.48

## Discussion

This study was designed and then evaluated for its psychometric properties using the CCS in family caregivers of people with AD. The results showed that the self-report CCS in family caregivers of people with AD had good reliability and validity. The scale presents both general and context-specific challenges that the caregiver face. In Iran, Abdollahpour et al. (29) developed a questionnaire to assess the caregiver burden for caregivers of people with dementia, based on literature review and expert opinion. They assessed the content validity and reliability (29). The present study was carried out because of the lack of a valid and reliable tool according to Asian and Iranian cultures. Having context-based information is essential for designing interventions to reduce stress and promote the wellbeing of family caregivers (40).

After designing and assessing face and content validity, a 10-item two-factor structure questionnaire was developed based on exploratory factor analysis. Factor 1 (effective role-play challenge) included items concerning difficulties related to communication with the patients, control of unusual behaviors, patient anxiety, and lack of information. This dimension focuses on the proper performance of the caregiver with respect to people and care situations. It assesses the challenges that caregivers are faced with in solving problems related to the patients' behaviors and the ability to manage symptoms of the patients. Therefore, this domain seems to be related to the caring ability of the caregivers (41). The second factor (lack of social–financial support) has items concerning the cost and supportive challenges for managing and caring for patients with AD such as lack of insurance coverage, loss of job while providing care, lacking cooperation from relatives, and lack of social support. All these factors expose the caregivers to challenges. This dimension is related to the financial burden that caregivers face during the caregiving process and the lack of social and formal support.

The caregiver burden questionnaire developed by Abdollahpour et al. (29) has 33 items and no subscale. The present study has similarities in terms of difficulties with patients' behaviors and the support of others. Our questionnaire includes considerations about job threats, insurance coverage, and limited social support, which were not included in the questionnaire by Abdollahpour et al. (29). The cost of care and the limited coverage of insurance, facilities, and support are more significant for Iranian caregivers of people with AD than other caregivers in other developing countries (28). Many tools on caregiver burden originate from developed countries and do not focus on these important issues. Because the questionnaire of Abdollahpour et al. is based on a literature review, it is reasonable that the financial issues are addressed only by one item in their questionnaire, and social support and insurance coverage were not included.

Gerritsen and Van der Ende developed a scale to measure the caregiving burden in the spouses of patients with dementia, which includes 13 items and two dimensions of “relationship” and “personal consequences” (42). Their scale and the CCS both address the difficulty of communicating with the patients, but the scale of Gerritsen and Van der Ende also addresses issues such as the caregivers' health, lack of personal time, and feelings of depression, anger, frustration, and embarrassment about the patients. These items are not present in the CCS. On the other hand, our tool addresses topics such as lack of information about care, the threat to job security, and the limitation of financial and social support, which were not included in Gerritsen and Van der Ende's study.

One well-known caregiver burden assessment tool, which was first developed for people with senile dementia, is the Zarit Burden Interview. Similar to the present questionnaire, the original version of this tool has 29 items and no subscales and covers issues such as support of relatives and others, embarrassing behaviors of the patients, and the cost of care (27). Still, aspects such as social support, insurance issues, and knowledge deficit of the caregivers about how to care are included in the present questionnaire, which were not addressed in the Zarit Burden Interview. In another study, Taemeeyapradit et al. developed a caregiver burden scale for patients with dementia based on literature review, interviews, and expert opinion in Thailand (28). Their scale has 18 items with three physical, psychological, and financial burden factors. The financial burden of care, lack of cooperation of others and relatives as caregivers, difficulties in communication with people and management of their behaviors, and lack of information and social support are similar to our CCS and to the scale by Taemeeyapradit et al. These extensive similarities may be due to the cultural similarities of the caregivers participating in both studies. But in our questionnaire, the caregivers' threat of losing their job was addressed, which was not included in the scale by Taemeeyapradit et al.

Conversely, the Zarit Burden Interview (27) and the Thai caregiver of people with dementia burden scale (28) include items concerning anxiety and depression, which were not included in our questionnaire. Some studies showed that Iranian caregivers of people with AD and dementia experience low to moderate anxiety and depression (43, 44). Also, since the purpose of this scale was to identify the challenges of caregivers and not the burden of care, the participants were more likely to point out the factors that led to the challenge in the qualitative stage; thus, the final scale did not have items about anxiety and depression.

Some contextual and cultural issues influenced item generation in this study. In Iran, like in many other developing countries, there are no formal institutions to support family caregivers. Also, community-based client care programs are in their infancy, and most care tasks for patients with AD are the responsibility of the patients' family, even if they do not receive special financial support (45). On the other hand, in Iranian and Eastern cultures, caring for a family member is one of the important values. Spouses and children are obligated to care for their relatives, even if they are not in a good financial situation or do not have sufficient facilities. Therefore, many of the items in our questionnaire focus on financial issues and social support to help identify the challenges faced by family caregivers in countries with low and moderate financial and social support systems.

## Implication

This scale can be used in research studies to assess and quantify the level of challenges in caregivers and can also be used to evaluate the effectiveness of various interventions that are aimed at decreasing the challenges faced by caregivers of people with AD. The CCS can identify where the support is most needed and who needs the support. Additionally, the CCS can also provide insights into which challenges are most frequently faced by the caregivers.

## Study limitation

This study has some limitations. Since the majority of the participants in this study were daughters of patients, and the real composition of caregivers in Iran is unknown, the generalization and representativeness of the findings could potentially be an issue. Another limitation of this study was related to the questions of the interview. Since the purpose of this study was to understand the family caregivers' challenges, the questions may have only led participants toward negative aspects of care, and they may have only remarked on problems in their care, while some caregivers may be satisfied with the care they provide and find caring for a loved one rewarding. It is a limitation that this aspect of caregiving was not captured. Another limitation of this study is the close relation between care burden and care challenge. Therefore, we suggest further testing of the relationship between the newly developed Care Challenge Scale and care burden among family caregivers of people with dementia to show the theoretical relevance.

## Study strength

The salient strength of this study is the application of Horn's parallel analysis and the exploratory graph analysis approach for the determination of factors. The final scale had 10 items, and because of the limitations that caregivers face, such as the limitation of time, a brief scale is ideal for assessing their challenges. The assessment of convergent and discriminant validity, calculation of McDonald's omega coefficient other than Cronbach's alpha, and evaluation of absolute reliability by calculation of standard error of measurement (SEM) are additional strengths of this study.

## Conclusion

This study showed that family caregivers of people with AD have two main challenges including the inability to carry out their caregivers' role effectively and a lack of financial and emotional support. Furthermore, the CCS has good validity and internal consistency and can reliably be used by healthcare professionals and researchers for evaluating family caregivers' challenges when designing and evaluating effective interventions to reduce their challenges.

## Data availability statement

The raw data supporting the conclusions of this article will be made available by the authors, without undue reservation.

## Ethics statement

The studies involving human participants were reviewed and approved by Mazandaran University of Medical Sciences Research Ethics Committee (IR.MAZUMS.REC.1401.13880). The patients/participants provided their written informed consent to participate in this study.

## Author contributions

HS, LH, and MA contributed to the conceptualization and design of the study. LH collected the data. HS and LH analyzed and interpreted the data. HS, LH, MA, ES, and SH prepared the manuscript draft. All authors contributed to the article and approved the submitted version.

## Conflict of interest

The authors declare that the research was conducted in the absence of any commercial or financial relationships that could be construed as a potential conflict of interest.

## Publisher's note

All claims expressed in this article are solely those of the authors and do not necessarily represent those of their affiliated organizations, or those of the publisher, the editors and the reviewers. Any product that may be evaluated in this article, or claim that may be made by its manufacturer, is not guaranteed or endorsed by the publisher.
